# Dipole-wind interactions under gap wind jet conditions in the Gulf of Tehuantepec, Mexico: A surface drifter and satellite database analysis

**DOI:** 10.1371/journal.pone.0226366

**Published:** 2019-12-23

**Authors:** Mauro W. Santiago-García, Alejandro F. Parés-Sierra, Armando Trasviña

**Affiliations:** 1 Department of Physical Oceanography, CICESE, Ensenada, Baja California, Mexico; 2 CICESE, Unidad La Paz, La Paz, Baja California Sur, Mexico; University of Guam, GUAM

## Abstract

Gap wind jets (Tehuano winds) trigger supersquirts of colder water and mesoscale asymmetric dipoles in the Gulf of Tehuantepec (GT). However, the effects of successive gap wind jets on dipoles and their effects inside eddies have not yet been studied. Based on the wind fields, geostrophic currents, and surface drifter dispersion, this research documented three dipoles triggered and modified by Tehuano winds. Once a dipole develops, successive gap wind jets strengthen the vortices, and the anticyclonic eddy migrates southwestward while the cyclonic eddy is maintained on the east side of the GT. During the wind relaxation stage, the cyclonic eddy may propagate westward, but due to the subsequent re-intensification of the Tehuano winds, the vortex could break down, as was suggested by surface drifter dispersion pattern and geostrophic field data. The effect of the Tehuano winds was evaluating via eddy-Ekman pumping. Under Tehuano wind conditions, Ekman downwelling (upwelling) inside the anticyclonic (cyclonic) eddies may reach ~ -2.0 (0.5) m d^-1^ and decrease as the wind weakens. In the absence of Tehuano winds, Ekman downwelling inside the anticyclonic eddy was ~ 0.1 (-0.1) m d^-1^. The asymmetry of downwelling and upwelling inside eddies during Tehuano wind events may be associated with Tehuano wind forcing.

## Introduction

**Fig 1:**
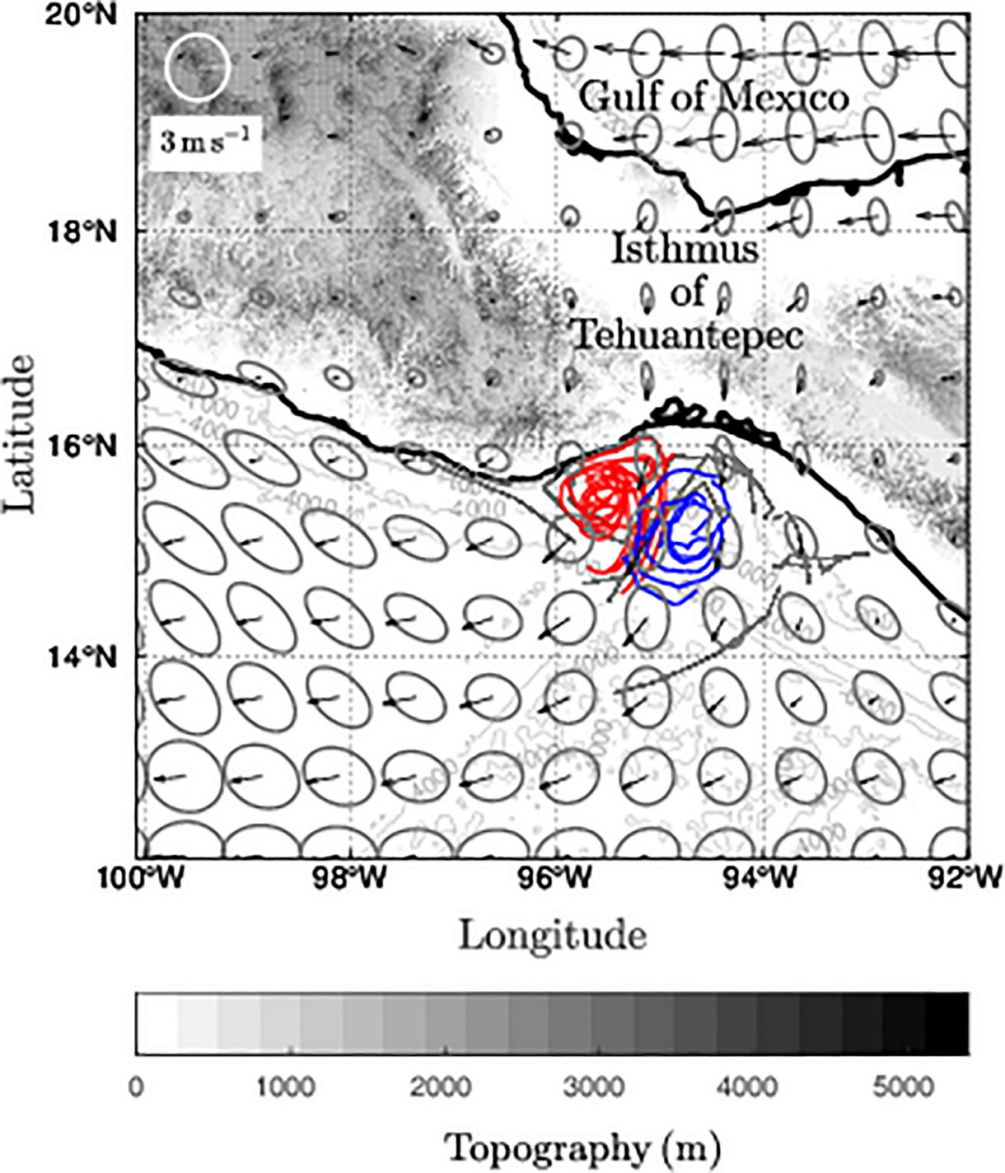
Dipolar circulation traced by surface drifters during weak Tehuano conditions. Red (blue) trajectories show an anticyclonic (cyclonic) eddy. Vectors show the average wind field and the associated variance ellipses for 20 days (from 25 June to 15 July 2000). The gray scale (solid grey lines) shows the topography (bathymetry) of the region (m) from GEBCO dataset (GEBCO_08 Grid, version 20100927: http://www.gebco.net). Drifter data was obtained from the Lagrangian experiment performed by Trasvina and Barton [9] and the wind from the Cross-Calibrated Multi-Platform (CCMP).

The Gulf of Tehuantepec (GT) is one of the few regions in the world with intense and intermittent gap wind jets that trigger ocean surface cooling [1,2] and asymmetric mesoscale circulation [3,4]. In the GT, successive gap wind jets, known as Tehuano winds or Nortes, are present throughout the year. Tehuano wind frequency and intensity are greatest during autumn and winter due to the passage of cold fronts coming from the midlatitudes and to a high-pressure system that forms in North America and moves southeastward over the Gulf of Mexico (GoM). These synoptic scale atmospheric conditions result in a pressure gradient between the GoM and the GT, generating airflow that is blocked by the mountains of the Sierra Madre but channeled through a mountain gap in the Isthmus of Tehuantepec [5,6] and the wind blowing over the Gulf of Tehuantepec (Fig 1). During summer, Tehuano winds are associated with the westward elongation and intensification of the Azores-Bermuda high-pressure center [7].

On average, Tehuano winds present speeds of ~20 m s^-1^ (maximum 35 m s^-1^) and last from 2 to 6 days with ~10 days between events [5,8]. Tehuano winds are stronger in winter than in summer. Although Tehuano winds are weaker in summer, they may still trigger dipolar circulation [9,10]. Satellite images show that successive Tehuano wind events generate surface cooling in the GT (~ -8.0°C) that occurs over a comma-shaped area, emerging from the coast and extending more than 300 km offshore, where it traces the periphery of the anticyclonic vortex that develops to the west of the axis of the wind [1,2].

The first hydrodynamic measurements of upper ocean responses to Tehuano winds were carried out in the winter of 1989 [3,4]. The survey showed that Tehuano winds induce an asymmetric response: a large and intense anticyclonic eddy is triggered on the western side of the GT, while a small and weak cyclonic eddy was found to develop in the eastern portion of the gulf. These features were detected by sea surface dynamic topography (relative to 250 dbar) that indicated a surface elevation anomaly (> 20 cm) associated with the anticyclonic eddy. In contrast, little evidence of the cyclonic eddy has been collected, as was observed by Barton et al. [3] for the winter of 1989. Although dipole triggering has also scarcely been documented during summer, surface drifter [10] and high-frequency radar [9] data have indicated that the dipole is only present for a few days.

The generation of eddies in the GT is not only due to Tehuano winds; eddies can also be produced by barotropic and baroclinic instabilities in the mean flow [11,12]. Other possible mechanisms of generation include instabilities in coastal currents due to the propagation of coastal Kelvin waves [13]. However, high-frequency wind forcing is arguably the main factor that generates coastal and mesoscale variability [9] and triggers dipoles in the GT [9–12,14].

Dipoles in the GT are associated with Tehuano winds and Ekman pumping [12,14]. The fundamental dynamics of dipole circulation are explained by Ekman pumping associated with wind stress that produces asymmetric transport under the axis of the wind, *i*.*e*. a convergence (divergence) to the right (left) of the downwind direction that results in a downwelling (upwelling) of the pycnocline and the geostrophic generation of an anticyclonic (cyclonic) eddy [12]. Both anticyclonic and cyclonic eddies may propagate from the coast in southwestward or westward directions, respectively. However, little evidence exists regarding the presence of cyclonic eddies in the GT based on direct observations, satellite databases, or numerical models.

Eddy statistics show that the development of vortices in the GT occurs mostly during autumn, winter, and spring [15,16]. Although there is little documented evidence, vortices have also been found to develop in summer [9,10]. The anticyclonic eddies have been found to be more numerous, intense, and long-lived compared to the cyclonic eddies [15–17]. Palacios and Bograd [17] reported the generation of between 2 and 6 (an average of 3.5) anticyclonic eddies per year. Zamudio et al. [13] showed similar results using satellite altimetry data and numerical modeling. The only contrasting results were reported by Gonzalez-Silvera et al. [16] who found more cyclonic than anticyclonic eddies. They reported 14 eddies with radii between 50 and 225 km, most of which were cyclonic eddies, based on satellite observations from November 1998 to March 1999. However, some vortices may not be associated with Tehuano winds given their regions of formation (<12° N). Eddy generation presents interannual variability, and eddies are more numerous, intense, and longer lasting during El Nino years compared to that of La Nina years, which has been associated with flow instability caused by the propagation of coastal Kelvin waves [13]. Typically, eddies have radii between 90 and 250 km, tangential velocities that may exceed 1 m s^-1^, and translation speeds between 9 and 16 km d^-1^ [15,16].

Some features of the GT may be key factors that attenuate cyclonic eddies, such as entrainment processes [18], the buoyant flow on the east coast of the gulf that may inhibit eddy production [19], and continental shelf interactions [20]. However, under certain conditions, cyclonic eddies develop and propagate. Based on satellite images, Müller-Karger and Fuentes-Yaco [15] suggested that cyclonic vortex formation occurs most likely to occur when a period of short and strong winds is followed by a period of low-intensity winds. Trasviña and Barton [10] used surface drifters to document dipolar structures in the presence of Tehuano winds and found that when the cyclonic eddy eroded, most of the cyclonic eddy drifters were incorporated to the anticyclonic eddy.

Although successive wind gusts are associated with the formation of eddies in the GT, the dipole evolution and the effects of Tehuano winds inside eddies via eddy-Ekman pumping are scarcely documented. This study assessed the velocity of Ekman pumping in Tehuano wind-triggered dipoles, and the roles played by both linear and nonlinear Ekman pumping components inside eddies were evaluated under Tehuano wind conditions.

This study focused on evaluating GT dipoles as persistent structures that are triggered and influenced by a set of Tehuano winds. Based on the dispersion pattern of the surface drifters and eddy-wind interactions, we show that once the dipole is generated, Tehuano winds may promote cyclonic eddy attenuation and strengthen the anticyclonic eddy in the GT. In contrast, once the dipole is generated, it propagates in the absence of Tehuano winds, with the anticyclonic and cyclonic eddies travelling southwestward and westward, respectively. The cyclonic vortex, in its journey to the west, interacts with the coastline, tends to attenuate, and disappears. The findings of this study contribute to understand of the dynamics of the GT, where Tehuano wind effects play a crucial role in sub-surface water pumping, favoring biological productivity [16,21–23] and the ocean-atmosphere flux of CO_2_ [24].

## Methods

A set of satellite and surface drifter data were used to evaluate the influence of Tehuano winds on the formation and evolution of dipoles in the GT. The vortices were identified from geostrophic fields using an eddy detection scheme based on velocity vector geometry [25]. Wind effects inside eddies were assessed using Ekman dynamics as described below.

### Wind and surface current fields

Wind data were obtained from the Cross-Calibrated Multi-Platform (CCMP) Ocean Surface Wind Velocity Product for Meteorology and Oceanographic Applications [26]. The spatial (temporal) resolution of the product was 0.25 x 0.25° (6 h). Surface current data were obtained from the GEKCO database [27] that was developed at the Center for Topographic Studies of the Ocean and Hydrosphere (CTOH). The velocity fields included geostrophic and Ekman components. The geostrophic component was derived from AVISO altimetry, and the Ekman currents were estimated from wind data from the QuikSCAT satellite [28]. The spatial (temporal) resolution of the product was 0.25º (daily).

### Tehuano winds

In this study, we define a Tehuano as wind coming from the north (*τ*_*y*_>1.5*τ*_*x*_) with speeds greater than > 6 m s^-1^ that persists for more than two days. These criteria were assessed with daily average winds obtained from the region of maximum Tehuano wind intensity (~94–95°W, 15°N).

### Surface drifters

**Fig 2:**
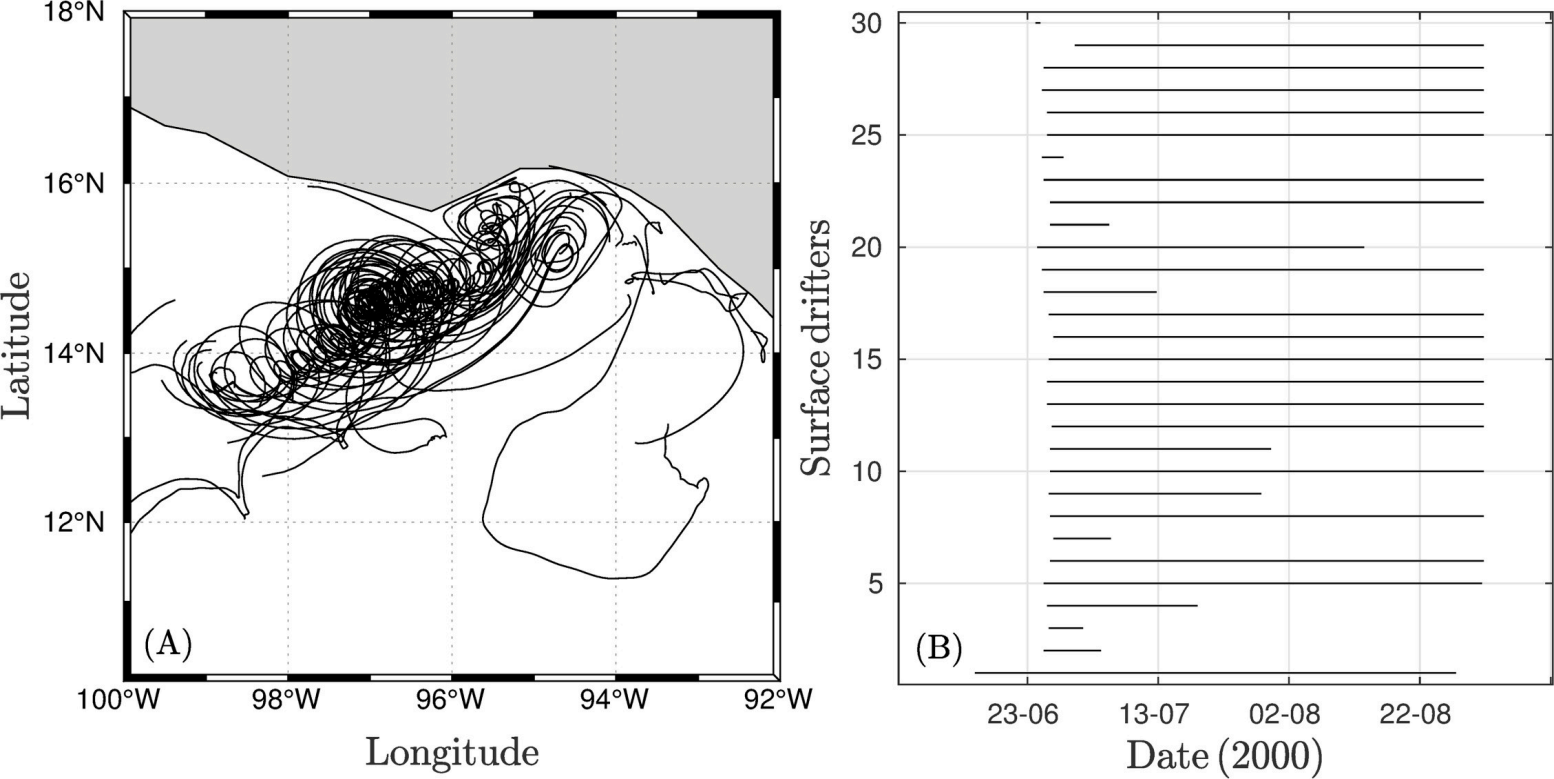
Surface drifter set. (A) Trajectories of 30 drifters released in the Gulf of Tehuantepec and (B) their lifetimes.

For this study, we used surface drifter data from the Lagrangian experiment performed by Trasviña and Barton [10] in June 2000. The Lagrangian experiment was designed to characterize the Costa Rica Coastal Current in the GT. Thirty surface drifters were drogued at 15 m and were released in the GT (Fig 2). Position data were acquired and processed by the Drifting Buoy Data Assembly Center (DAC) following the methodology described by Hansen and Paulin [29]. The position and velocity of the drifters were recorded every 6 h. For further details of the experimental procedure, please review Trasviña and Barton [10]. To remove the inertial signal, drifter positions were filtered following the methodology described by Sudre et al. [27], and the positions were smoothed over a 72-h window and speed and direction were computed from two consecutive positions at six-hour intervals.

### Eddy detection

From surface current data obtained from the GEKCO database, eddy detection was performed using the algorithm developed by Nencioli et al. [25]. This method is based on four constraints: (1) a sign change of the meridional velocity (v) along an east-west section, (2) a reversal zonal velocity (u) on a north-south section, (3) a local minimum of the speed at the eddy center, and (4) a constant sense of rotation along the four quadrants of the eddy. The edge of the eddy was defined as the outermost closed stream function around the center. The algorithm also identified the trajectory of the eddy. For a further detailed review, see Nencioli et al. [25]. The dipolar structure and its evolution were analyzed along the eddy trajectories to quantify eddy features, such as kinetic energy, relative vorticity, translation speed, size, and Ekman pumping. Only eddies with radius > 40 km were considered.

### Ekman pumping

The effect of the wind on the thermocline depth can be obtained via the Ekman pumping velocity (W_tot_). The relative vorticity (ζ) of ocean eddies is not much smaller than the Coriolis parameter (f). For eddies of the GT, the Rossby number (Ro = ζ/f) is ~ 0.2 [30]. Therefore, Ekman pumping depends on the total vorticity [31,32]. Ekman pumping was estimated following the methods of Gaube et al. [33] with the linear and nonlinear components as follows:

$$W_{tot}=W_{c}+W_{\zeta }$$ (1)

$$W_{c}=\frac{1}{\rho (f+\zeta )}\left(\frac{\partial \tau_{y}}{\partial x}-\frac{\partial \tau_{x}}{\partial y}\right)$$ (2)

$$W_{\zeta }=\frac{1}{\rho {\left(f+\zeta \right)}^{2}}\left(\tau_{x}\frac{\partial \zeta }{\partial y}-\tau_{y}\frac{\partial \zeta }{\partial x}\right)$$ (3)

where *ρ* = 1020*kgm*^−3^ is the surface water density, *f* = 2*Ωsin*(*ϕ*) is the Coriolis parameter for latitude *ϕ*, *Ω* is the rotation rate of the earth, *τ*_*x*_ and *τ*_*y*_ are the zonal and meridional components of wind surface stress, and *ζ* is the vertical vorticity component.

Wind stress was estimated as the difference between wind speeds and ocean currents, using the bulk formula:

$$\tau =\rho_{a}C_{D}(u_{a}-u_{o})|u_{a}-u_{o}|$$ (4)

where *ρ*_*a*_ = 1.2*kgm*^−3^ is the air density, which is assumed to be constant; *C*_*D*_ is the drag coefficient as defined by Ocampo-Torres et al. [34]; *u*_*a*_ is the CCMP daily wind average at 10 m; and *u*_*o*_ is the surface current velocity.

Including the effects of the surface currents in wind stress calculations produces variation in Ekman flux (convergence and divergence) and therefore in the Ekman pumping [35,36]. Eddy-wind interaction has been analyzed under a uniform wind blowing over an idealized eddy; wind stress was enhanced on the side of the vortex where its velocity is opposite to that of the wind, while wind stress was reduced on the other side where the wind and currents traveled in the same direction [33, 35–37].

Under conditions of a uniform wind blowing over a symmetric eddy, the eddy-wind interaction imprints a curl unto the wind stress, which has a polarity opposite to that of the vorticity of the surface current of the eddy. This results in eddy attenuation by inducing Ekman upwelling in the center of the anticyclonic vortices [35,36,38] and Ekman downwelling in cyclonic eddies [33]. Recently, Lu et al. [39] showed that the eddy-shape and wind direction impact in the Ekman pumping inside anticyclonic eddy. The Ekman pumping arises from the curl of the surface stress is known as linear Ekman Pumping (W_c_). The eddy surface vorticity is another factor that impacts on the Ekman pumping. The interaction between the wind stress and the vorticity gradient of the surface current generates a dipole of Ekman upwelling and downwelling inside mesoscale eddies, known as the non-linear Ekman effect (W_ζ_). The location of upwelling and downwelling cells depends on the pattern of the wind [37]. The net impact of the upwelling/downwelling movement inside eddies was evaluated using the azimuthal average of the Ekman pumping components. Finally, Ekman pumping components were integrated inside the vortex as follows:

$${\left(W_{c},W_{\zeta },W_{tot}\right)}_{eddy}=\frac{1}{A}\int_{A}(W_{c},W_{\zeta },W_{t})dA$$ (5)

where A is the eddy area. Therefore, the results represent global Ekman pumping inside the eddy.

To separate large and mesoscale features of Ekman pumping, a spatial Hanning filter with a cutoff length scale of 6° longitude by 6° latitude was applied to the direct estimate of Ekman pumping. Mesoscale W_tot_ variability was computed by removing low-pass filters from the original database.

## Results

### Dipole features: Surface drifters and geostrophic currents

The formation of three Tehuano wind dipoles is described in three stages: before, during, and after the development of dipoles.

**Fig 3:**
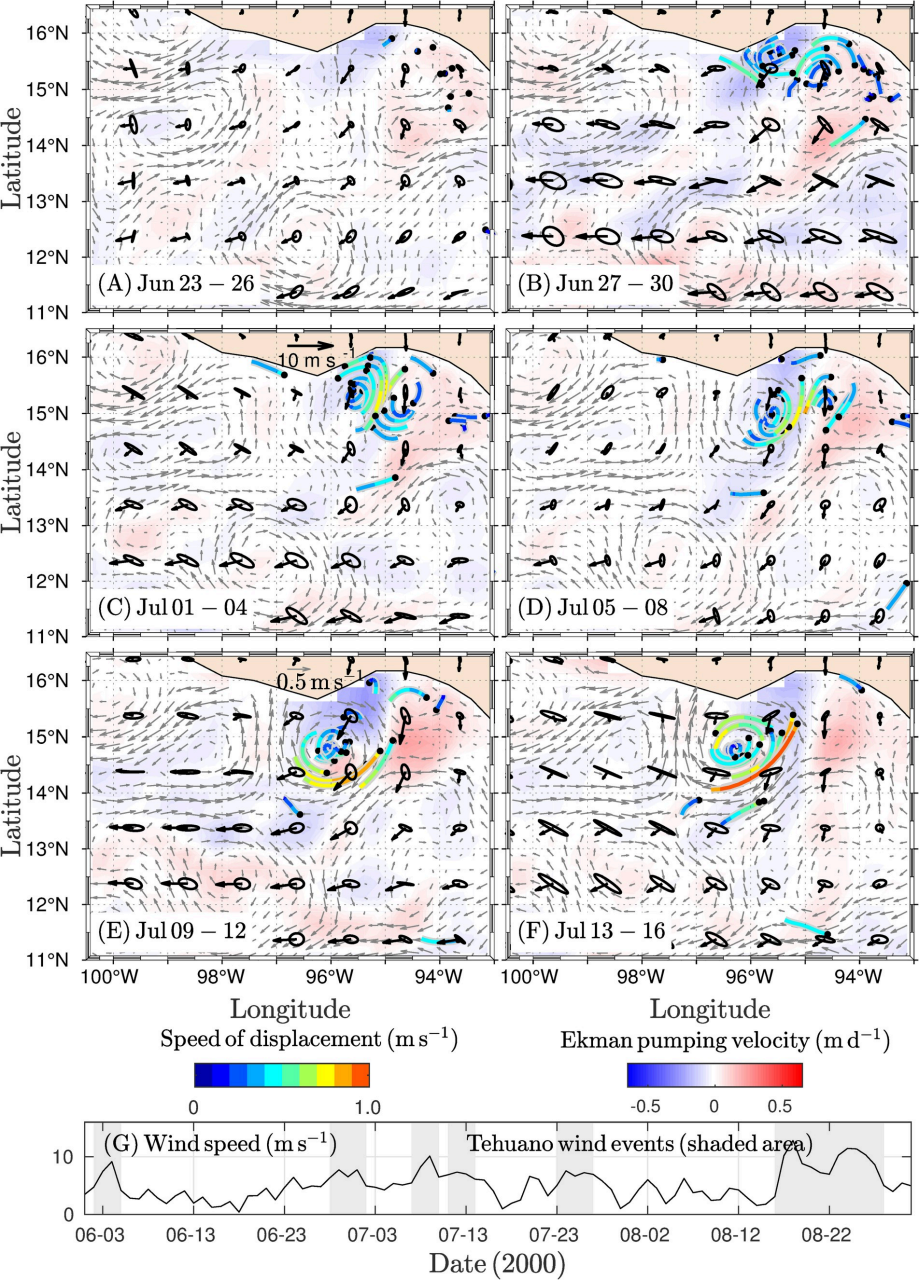
**Four-day average of the wind (black vectors), geostrophic current (gray vectors), and Ekman pumping velocity (W**_**tot**_**) fields (red-blue scale) for 24 days covering periods of the development and erosion of the first dipole traced mainly by drifter trajectories.** The variability ellipses were computed from the standard deviation of the wind velocity components. (A) Black dots indicate the initial drifter positions, with most of the drifters starting on 26 Jun. (B) Dipole development under Tehuano wind conditions. (C) The cyclonic side of the dipole begins to erode. (D) The erosion of the cyclonic side of the dipole is complete. (E-F) The anticyclonic eddy strengthens. (G) Wind speed along a section of the GT (~94–95°W, 15°N). Tehuano wind events (shading) and vertical dashed lines indicate periods corresponding to the panels (A-F). Drifter speed is shown in m s^-1^.

The first dipole was triggered under weak cyclonic circulation conditions, and circulation favored anticyclonic circulation (elongated with SW-NE orientation) located in the SE potion of the GT (S1 Fig). Under these conditions, Tehuano winds onset and strengthened cyclonic circulation in the eastern region of the gulf, while the development of anticyclonic circulation with a S-N orientation was evident in the western region (Fig 3A and 3B). The cyclonic portion of the dipole was located over the eastern and central regions of the GT (Fig 3B). The vortex dimension was a ~50 km radius based on drifter trajectories and the geostrophic field (Fig 3B), but the cyclonic vortex was short-lifetime (~4 days). Under conditions of persistent Tehuano winds, the drifters that traced the vortex were incorporated into the anticyclonic side of the dipole (Fig 3C–3E). This erosion of the cyclonic vortex agrees with the attenuation of the vortex in the geostrophic field (Fig 3B and 3C). In contrast, the anticyclonic side of the dipole was strengthened. The anticyclonic vortex formation stage was traced by the drifters (Fig 3B). The distribution and speed of the drifters indicated that the vortex increased in size and intensity during propagation (Fig 3C–3F). Some drifters reached velocities of ~1 m s^-1^ in the strongest wind events (Fig 3F). The drifters showing these magnitudes were located in the central gulf region.

**Fig 4:**
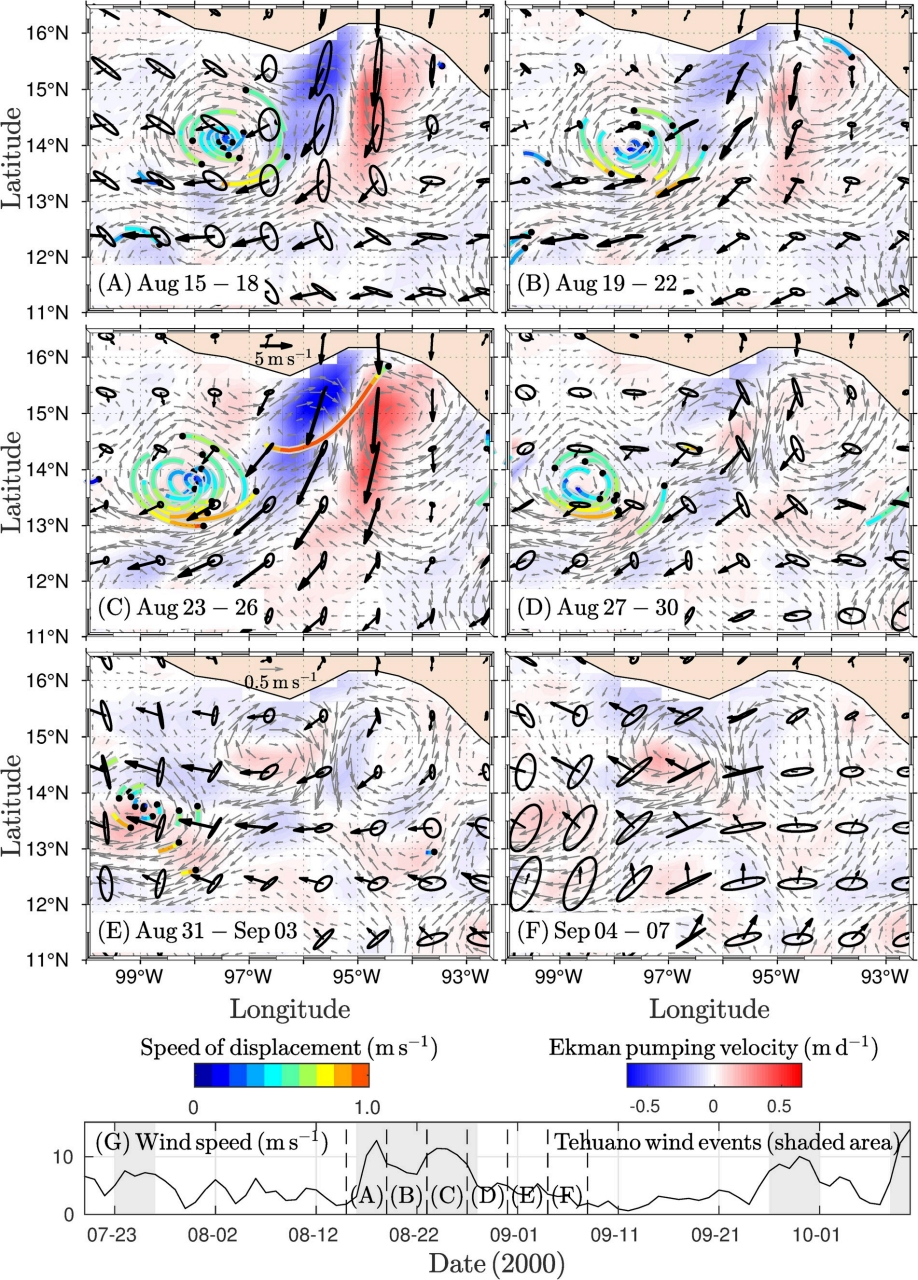
**Four-day average of the wind (black vectors), geostrophic current (gray vectors), and Ekman pumping velocity (W**_**tot**_**) fields (red-blue scale) for 24 days covering the triggering and propagation of the second dipole.** The variability ellipses were computed from the standard deviation of the wind velocity components. (A-B) Under Tehuano wind conditions, the anticyclonic eddy forms (~96° W, ~15° N) while the cyclonic eddy is maintained in the eastern portion of the GT. (C) The dipole develops, and one drifter traces it. (D) Toward the end of the Tehuano wind period, the dipole is fully developed. (E-F) The vortices of the dipole propagate. (G) Wind speed along a section of the GT (~94–95°W, 15°N). Tehuano wind events (shading) and vertical dashed lines indicate periods corresponding to the panels (A-F). The drifter speed is indicated in m s^-1^.

The second dipole developed under cyclonic circulation conditions and was apparently induced by two mesoscale anticyclonic vortices that were located offshore. These vortices generated a sinuous eastward flow that was deflected to the north upon reaching the eastern end of the GT, following the coast and supporting cyclonic circulation (S2 Fig). These circulation conditions prevailed when the average wind blew towards the southwest in the GT. Once the wind intensified, the cyclonic vortex strengthened, and the anticyclonic vortex tended to develop at ~96° W and ~14.5° N along the western coast of the GT (Fig 4A and 4B). The anticyclonic side of the dipole developed entirely during the stage of maximum wind intensity, and one drifter followed the periphery of the vortex (Fig 4C). The dipole persisted and then propagated in the absence of Tehuano winds (Fig 4D–4F), as is described in the next subsection.

**Fig 5:**
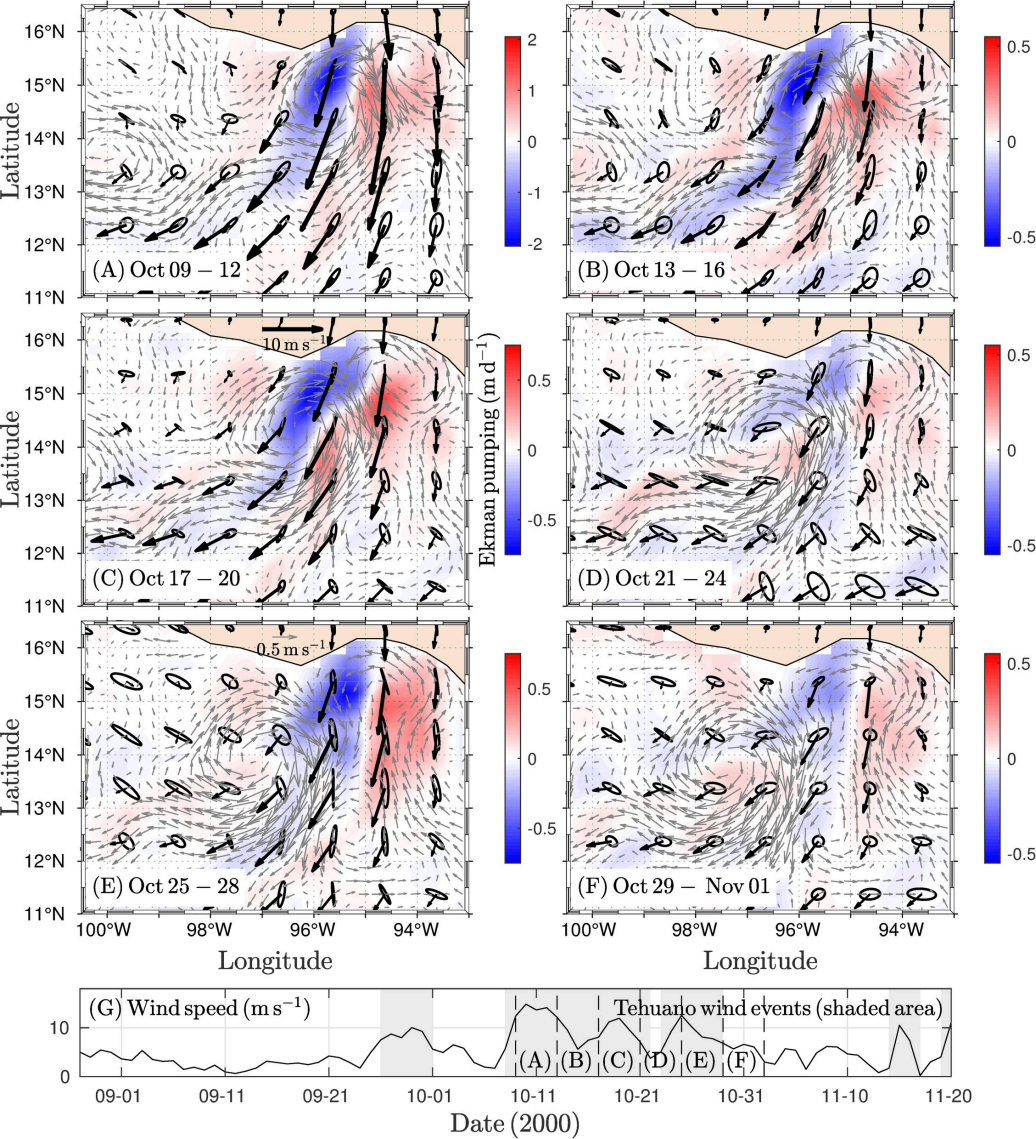
**Four-day average of the wind (black vectors), geostrophic current (gray vectors), and Ekman pumping velocity (W**_**tot**_**) fields (red-blue scale) for 24 days covering the triggering and evolution of the third dipole.** The variability ellipses were computed from the standard deviation of the wind velocity components. (A) Under stronger Tehuano wind conditions (~12 m s^-1^), the dipole is fully developed. (B-C) Tehuano winds are persistent, and the vortices are strengthened and propagated. (D) With minimum wind speeds, the anticyclonic eddy is located to the southwest of the GT while the cyclonic eddy covers most of the GT. (E) The wind re-intensifies, and closed cyclonic circulation breaks down. (F) After which, only the anticyclonic eddy persists. (G) Wind speed along of a section of the GT (~94–95°W, 15°N). Tehuano wind events (shading) and vertical dashed lines indicate periods corresponding to the panels (A-F). The drifter speed is shown in m s^-1^.

Under condition of pre-existing anticyclonic circulation, a third dipole triggered, which was promoted by the presence of a cyclonic vortex in the western portion of the GT. The influence of a Tehuano event on the GT re-intensified the anticyclonic vortex and initialized the development of the cyclonic vortex along the eastern coast (S3 Fig). The dipole was fully developed with the stronger Tehuano event (four-day average of ~12 m s^-1^; Fig 5A). In addition, winds were persistent and strengthened the vortices, and the anticyclonic eddy propagated southwestward while the cyclonic eddy was maintained in the east of the GT (Fig 5B and 5C). During wind relaxation periods, the cyclonic vortex tended to dominate the circulation of the GT, as shown in Fig 5D. As another Tehuano wind event developed, the cyclonic vortex broke down and disappeared (Fig 5E and 5F).

### Eddy features

The properties of the dipole vortices (anticyclonic eddy A1 and cyclonic eddy C1 for the first dipole and so on) are described in terms of the evolution of an integrated parameter, such as kinetic energy, relative vorticity, translation speed, or size. Eddy intensity was evaluated by the kinetic energy, relative vorticity, and size (vortex radius). The parameters were normalized by the maximum value recorded for each parameter.

**Fig 6:**
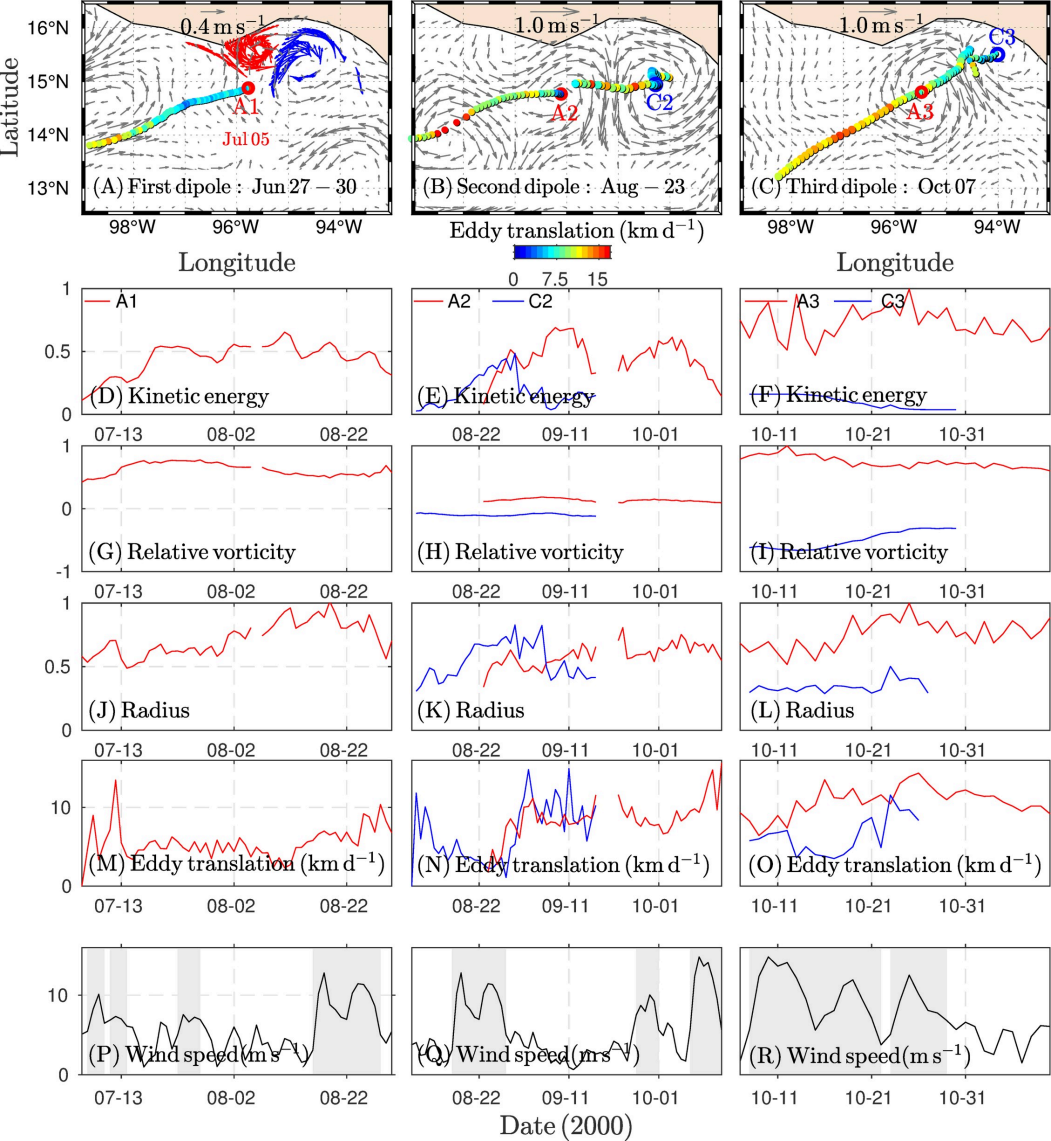
Dipole development and evolution in the GT: drifters and geostrophic currents. (A-C). Dipole eddies are indicated by the letter A (C) for anticyclonic (cyclonic) eddies followed by the corresponding dipole number. The first dipole was identified by visual inspection, based on the trajectories of surface drifters (red/blue vectors for A/C) and geostrophic currents, as is showed in A. The anticyclonic eddy was fully developed and detected by the Nencioli Algorithm on Jun 05. The second and third dipoles were detected by the Nencioli Algorithm on the indicated date. The properties of the A and C eddies are as follows: (D-F) kinetic energy, (G-I) relative vorticity, (J-L) size, and (M-O) eddy translation speed. (P-R) Wind speed along a section of the GT (~94-95° W, 15° N), the shaded areas indicate Tehuano wind periods. The maximum value of each parameter normalized the eddy properties.

The first dipole was detected by visual inspection, as described above. Only the anticyclonic eddy was detected by the Nencioli algorithm around July 05 (Fig 6A). The eddy A1 strengthened in the first stages of its lifetime when two weak Tehuano wind events occurred. The intensity slightly decreased around Aug 10, as indicated by the kinetic energy when the eddy moved away from the coast (Fig 6D).

The second dipole developed after a persistent Tehuano wind event with a bi-modal wind speed distribution and a relative minimum around 22 Aug (Fig 6Q). After the wind re-intensified, it detonated the formation of the anticyclonic eddy, and the dipole was structured (Fig 6B). Under Tehuano wind conditions, the vortices intensified, as shown by the kinetic energy (Fig 6E). Once the Tehuano winds ceased, the eddies propagated. Initially, both eddies travelled westward, but later the anticyclonic eddy propagated southwestward. However, the cyclonic eddy attenuated until it disappeared due to eddy-coastline interactions while the anticyclonic eddy freely propagated.

The third dipole developed from the presence of an anticyclonic eddy in the GT, which propagated towards the southwest (Fig 6C). The influence of the Tehuano winds on the gulf strengthened the anticyclonic vortex and triggered a cyclonic eddy on the eastern side of the GT on Oct 7 (S3 Fig). The cyclonic eddy was unstable, and the vortex broke during the Tehuano wind re-intensification stages but restructured during the relaxation stage. From the center eddy position, we used a linear interpolation to fill the gaps in eddy detection. In this case, the eddy radius was defined as 50 km. The anticyclonic eddy was generated and evolved under stronger Tehuano winds, as shown by the kinetic energy (Fig 6F) and the relative vorticity (Fig 6I). The anticyclonic vortex was consistently stronger, as shown by the kinetic energy (Fig 6F), relative vorticity (Fig 6I), and its larger size than that of the other eddies (Fig 6L).

Eddy translation speeds fluctuated between 8 and 15 km d^-1^. The anticyclonic eddy was faster under Tehuano wind conditions than under non-Tehuano wind conditions. For example, the translation speed of the anticyclonic vortex of the first dipole increased from ~8 to ~13 km d^-1^ (Fig 6M), and the same pattern was present for the third dipole (Fig 6O). On the other hand, the cyclonic eddy was slower under Tehuano wind conditions due to the constraint of the eastern coast of the GT (Fig 6B and 6C).

### Ekman pumping inside the dipoles

**Fig 7:**
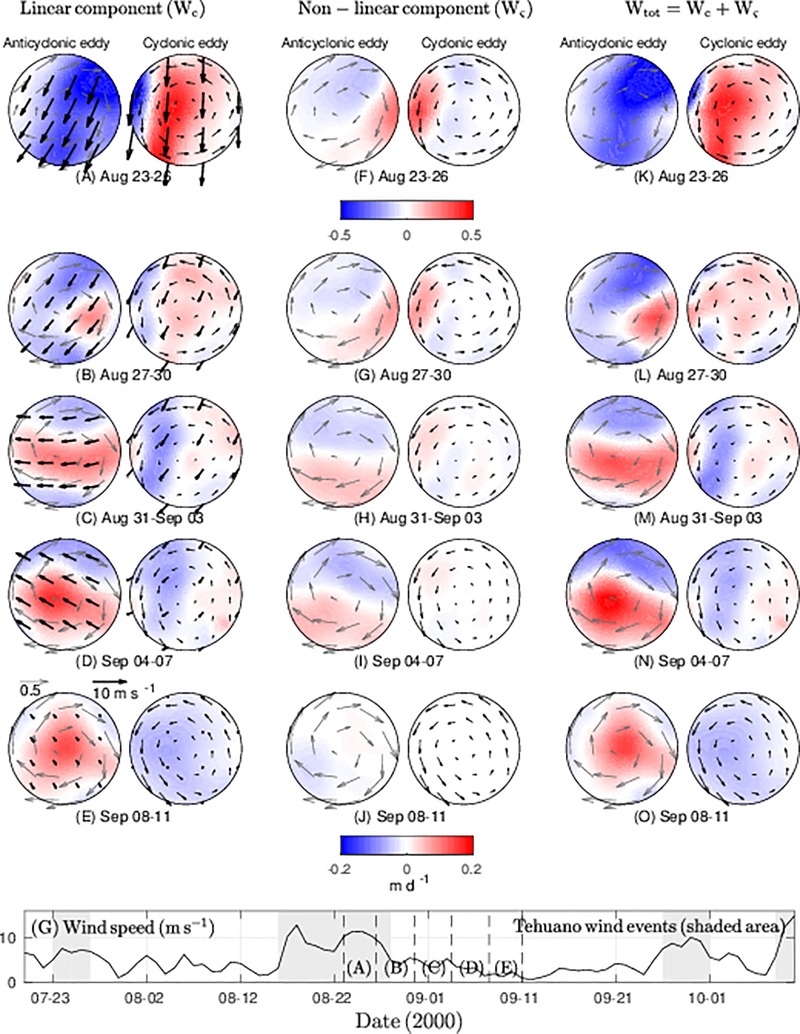
Vertical velocities inside the eddies of the second dipole are shown under Tehuano wind conditions. (A-E) The spatial structure for the linear and (F-J) non-linear components of Ekman pumping, and (K-O) the total vertical velocity inside eddies are shown in m d^-1^. The geostrophic (wind) field is shown with gray (black) vectors. The eddies have been normalized by their radii.

Dipoles were the main structures associated with the effects of Tehuano winds in the GT. In general, a downwelling zone was present on the west coast of the GT while an upwelling zone was present on the east coast that reached vertical velocities of ~ -2 and ~1 m d^-1^, respectively (see Fig 5). Under these conditions, dipoles may be developed in the GT. The role of the linear and non-linear Ekman pumping velocity components was assessed as a schematic picture of eddy-wind interactions for the second dipole, where the cyclonic vortex presented the longest lifetime compared to that of the other vortices and showed characteristic patterns pre- and post-Tehuano wind activity (Fig 7).

The linear Ekman pumping component produced downwelling that predominated during Tehuano wind conditions (Fig 7A and 7B), but when the wind ceased, upwelling predominated in the presence of the anticyclonic eddy (Fig 7C–7E). With regard to the cyclonic vortex, upwelling was dominant under Tehuano conditions and downwelling was dominant under post-Tehuano conditions. Under the influence of Tehuano winds, the non-linear component showed a bimodal anticyclonic eddy structure, and downwelling was located in the northern region of the vortex (Fig 7F and 7G). In the post-Tehuano period, the bimodal structure persisted but was of lesser magnitude (Fig 7H and 7I). For the cyclonic vortex, upwelling was dominant over the eddy and of greater intensity under Tehuano wind conditions.

**Fig 8:**
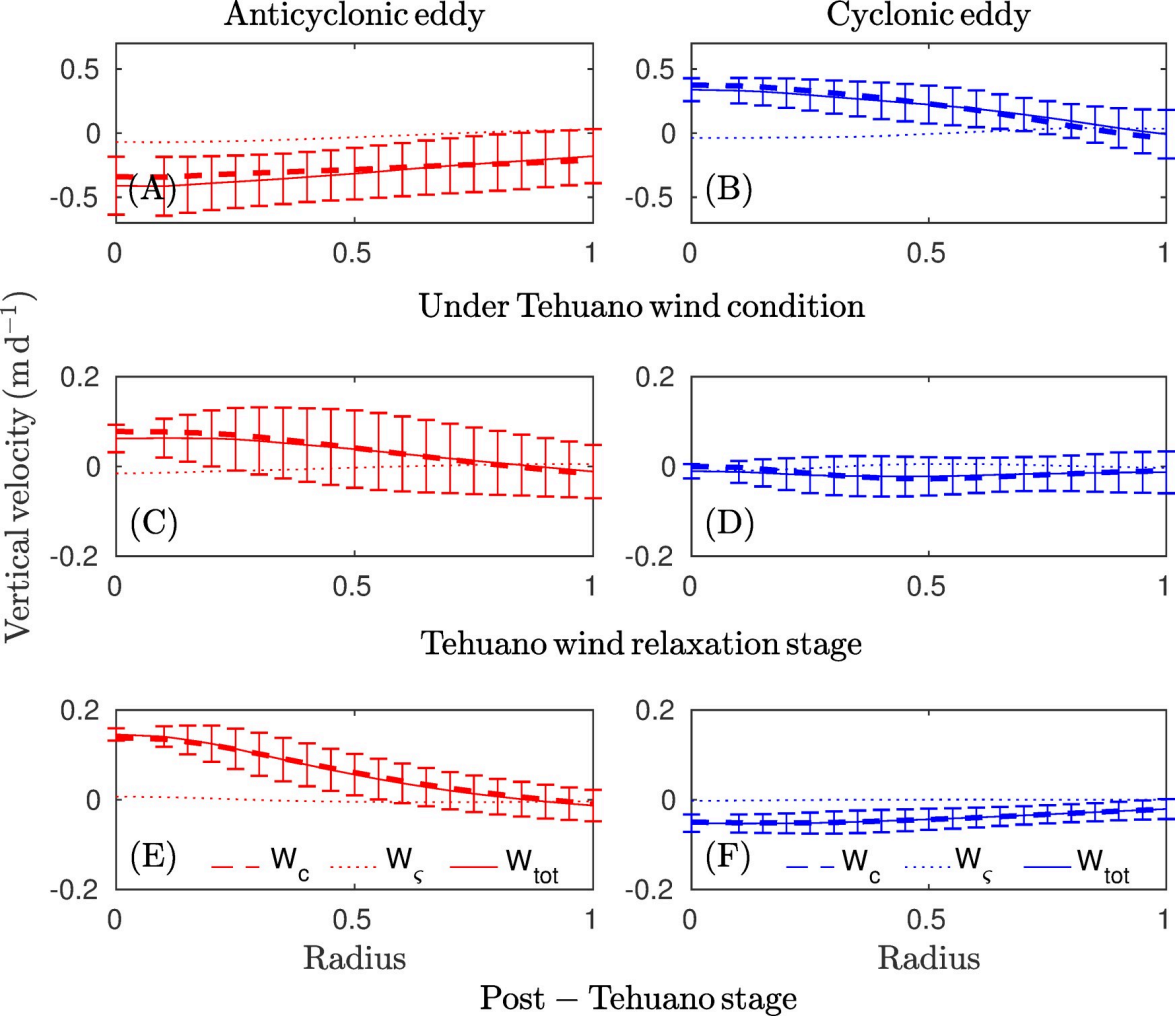
The azimuthal average for an anticyclonic (cyclonic) eddy is shown for every component of Ekman pumping for three Tehuano wind stages: (A-B) under the influence of Tehuano winds, (C-D) the wind relaxation period, and (E-F) post-Tehuano conditions (see Fig 4). Bars indicate the standard deviation for W_tot_.

Based on the structure and the magnitude of the Ekman pumping components, it was evident that the linear component was the main factor for total Ekman pumping (Fig 7K–7O). To evaluate the net impact of upwelling and downwelling movements inside the eddies, we used the azimuthal average of the Ekman pumping components for three stages: Tehuano wind conditions (Fig 8A and 8B), the wind relaxation period (Fig 8C), and post-Tehuano wind conditions (Fig 8D and 8E; see Fig 4). The results showed that the maximum vertical velocities were present in the core of the eddy and that the linear component was the main component in total Ekman pumping.

### Global Ekman pumping inside the eddy

**Fig 9:**
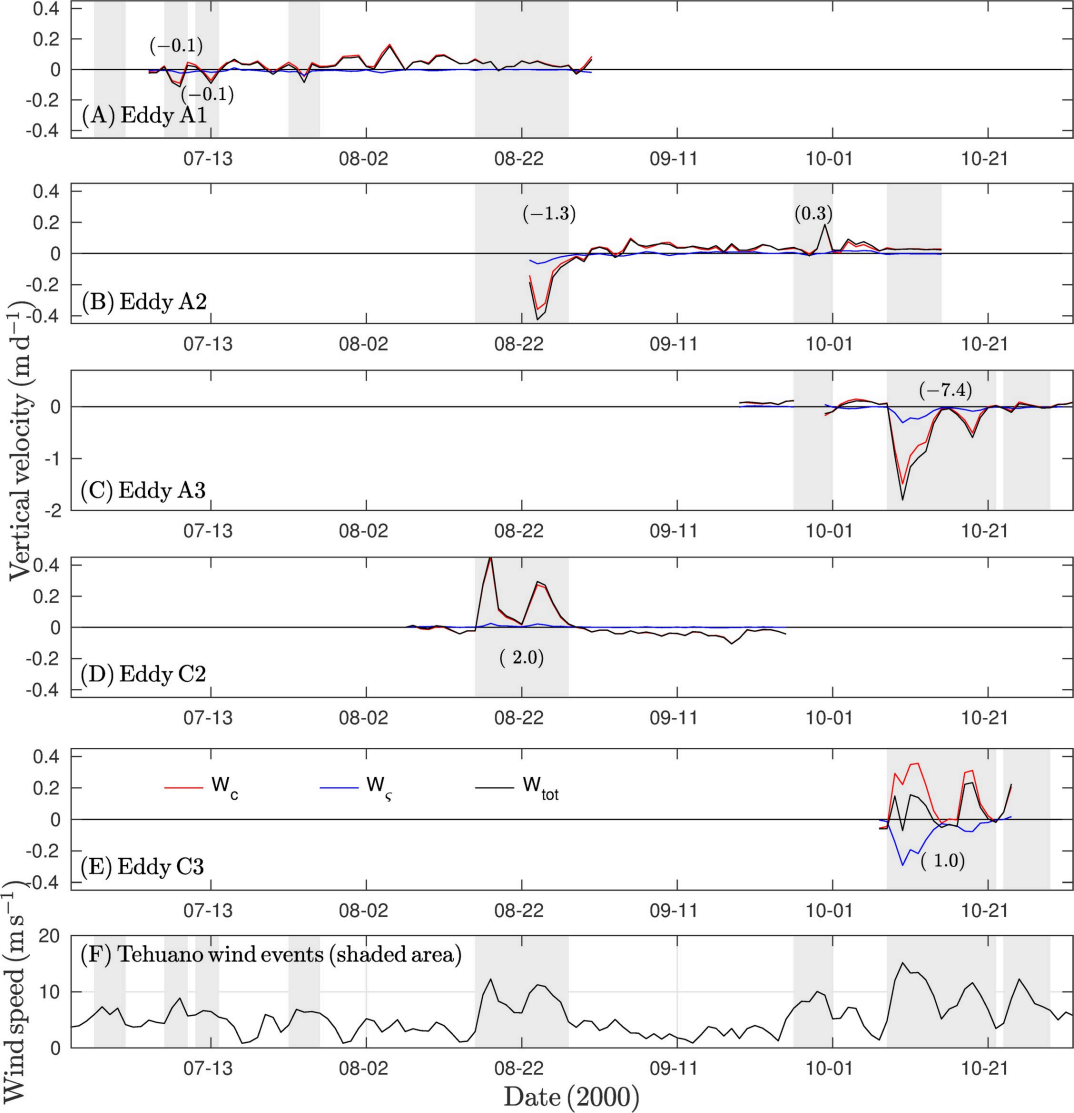
Global Ekman pumping inside eddies: (A) eddy A1, (B) eddy A2, (C) eddy A3, (D) eddy C2, and (E) eddy C3 as calculated from Eq 5. (F) Time series of the wind speed in the region of maximum intensity (~94–95°W, 15°N) where shaded areas indicate periods of Tehuano winds. The global Ekman pumping (W_tot_) inside eddies was estimated during the Tehuano wind event and indicated by parentheses.

Global Ekman pumping is a measure of the net vertical velocity inside an eddy (see Eq 5). In general, under Tehuano wind conditions, global Ekman pumping inside the anticyclonic eddy was negative (downwelling), while it was positive (upwelling) for the cyclonic vortex. Far from the influence of Tehuano winds or in the absence of Tehuano winds, the relationship inverted and was weaker (Fig 9).

Tehuano winds produced downwelling inside anticyclonic eddies. For eddies A1, A2, and A3, the net vertical velocity reached ~ -0.1 (Fig 9A), ~ -0.4 (Fig 9B), and ~ -2.0 m d^-1^ (Fig 9C), respectively. The maximum vertical velocities coincided with stronger winds (Fig 9F), which was evident for the A2 and A3 eddies. Once the Tehuano winds ceased, upwelling predominated inside the eddies (vertical velocity ~ 0.1 m d^-1^).

For cyclonic eddies during Tehuano wind conditions, upwelling was dominant within eddies. Vertical velocities reached ~ 0.4 m d^-1^ under conditions of stronger Tehuano winds (Fig 9D and 9E). The vertical speed decreased as the Tehuano wind weakened (Fig 9F). In the absence of Tehuano winds, the net vertical velocity was negative inside the eddies (~ -0.1 m d^-1^).

The effect of the Tehuano winds on the vertical displacement of the thermocline (i.e., Ekman pumping (W_tot_) inside the vortices) was evaluated by integrating the vertical flow during Tehuano wind periods for eddies. Vertical displacements reached ~ -7.4 m (downwelling) for the anticyclonic eddy and ~ 2 m (upwelling) for the cyclonic vortex (Fig 9).

## Discussion

Our study documents three dipoles that were triggered and modified by subsequent Tehuano wind events in the GT. The wind effects on eddies were assessed via Ekman pumping in three stages: during the Tehuano wind event, the wind relaxation period, and post-Tehuano conditions.

The formation of eddies in the GT has been documented with satellite images [1,2,15,16], direct observations [3,4,9], and numerical modeling [18,20,40]. However, research on the development and evolution of dipoles triggered by Tehuano winds is scarce. This work shows that Tehuano winds were capable of generating dipoles in circulation modulated by background flow, i.e., meanders and eddies (S1–S3 Figs). For example, the second dipole was triggered during conditions of general cyclonic circulation. With the onset of Tehuano winds, cyclonic circulation was confined to the east coast of the GT, while anticyclonic circulation was triggered on the west coast (S1 Fig). The third dipole developed analogously. The winds tended to intensify the anticyclonic vortex inside the GT as it propagated southwestward, while a cyclonic vortex developed on the west coast and was confined during Tehuano wind events, resulting in a dipole with a SW-NE orientation (S3 Fig).

The generation of the first dipole was previously documented by Trasviña and Barton [10] from the velocity fields of a set of drifters. They showed that dipole generation occurred during the intensification of Tehuano winds. After which, the anticyclonic eddy propagated while the cyclonic vortex dissipated. They assumed that the thickness of the mixed layer limited vortex development and that eddy-suction in the cyclonic eddy could expose the pycnocline to surface processes (e.g., mixing) and eventually dissipate the vortex. The results of our research suggest that vortex dissipation is associated with the direct effect of the Tehuano wind sequence on the cyclonic vortex.

The attenuation of the cyclonic vortex could be explained by two factors: the propagation of the vortex to the west and its exposure to subsequent Tehuano wind events. The results show that during a Tehuano wind event capable of detonating a dipole, the cyclonic vortex was confined on the east side of the GT while the anticyclonic vortex was present on the west side. Once the wind ceases, the vortices propagated; the cyclonic (anticyclonic) eddy westward (southwestward) due to the beta effect and self-advection [38,41]. The cyclonic eddy may move over the central region of the GT, as seen with the cyclonic vortices of the first (Fig 3B) and third (Fig 5D) dipoles. A subsequent Tehuano wind event generated a jet current in the central region of the gulf (i.e., on the cyclonic eddy). The jet may break down the cyclonic side of the dipole. Multiple and successive Tehuano wind events may intensify this effect. The features of the jet current are consistent with the assumptions described. Trasvina et al. [4] document that jet current is restricted to 40 or 50 m depth under Tehuano wind conditions (speed < 15 m s^-1^) and during the most intense Tehuano wind phase, the mixed layer has been found to deepened rapidly in the central region of the gulf, which is a process associated with shear-induced entrainment within the jet stream.

The dispersion pattern of the drifters inside the cyclonic vortex and the geostrophic field data support the mechanism described above. The cyclonic eddy, which was traced by the drifters, occupied the central zone of the GT (Fig 3B). Under conditions of persistent and weak Tehuano winds (~16 days; Fig 3B–3E), the drifters that traced the cyclonic vortex were incorporated into the anticyclonic vortex. At this stage, the drifters reached velocities of ~1 m s^-1^ in the central gulf region. In this region, a difference was present in the direction and magnitude of the trajectories of the drifters and the geostrophic currents (Fig 3D), which has been associated with small-scale processes inherent in the trajectory records of individual drifters [27].

The dissipation of the cyclonic vortex A3 also presented a similar pattern. This vortex developed during the most intense Tehuano wind event (Fig 5A). The wind persisted, and the vortex migrated westward at a speed of ~ 4 km d^-1^. During a period of low wind intensity, the vortex was located in the central region and the western side of the GT. Subsequently, a re-intensification of the winds occurred that coincided with the break-up of the vortex structure. This suggests that the sequence of Tehuano wind events may play an essential role in the attenuation of the cyclonic vortex in the GT, a phenomenon that has not yet been systematically studied.

Some features of the GT may be essential to the attenuation of the cyclonic eddy, such as entrainment processes associated with shallow thermoclines in the region [18]. Recently, Velazquez-Muñoz et al. [20] used a nonlinear, hydrostatic, and tridimensional model forced by a realistic wind field, which included three Tehuano wind events in an ocean at rest with horizontally uniform vertical stratification and actual bathymetry. Their model only reproduced an anticyclonic vortex. From a momentum balance analysis using the barotropic component, Velazquez-Muñoz et al. [20] found that the nonlinear terms of advection, acceleration, and bottom friction were important on the east coast and could inhibit the formation of the cyclonic eddy mainly due to the presence of the continental shelf. Another feature that has been found to suppress eddy production is the presence of a buoyant flow observed on the east coast of the gulf, as suggested by Barton et al. [19].

In the GT, little information exits on cyclonic eddies while the anticyclonic vortex has been widely documented. Our results show that the anticyclonic eddy strengthens under Tehuano wind events. This is evident in the structure traced by the drifters (Fig 3) and the anticyclonic vortices of other dipoles. For example, the intensity of anticyclonic vortex A3 was higher than that of the others and was associated with the production of negative vorticity due to the influence of stronger Tehuano winds (Fig 6). The tendency to produce relative negative vorticity in the gulf under Tehuano wind conditions has been observed in coastal circulation radar data [42]. The same mechanism has been proposed for Santa Barbara Bay, a region dominated by positive wind stress curl and by cyclonic eddies that are more intense than anticyclonic eddies, particularly on the surface [43]. For the GT, Chang et al. [30] found that the anticyclonic eddy was intensified by successive wind jets, based on sea level heights from satellite data and numerical modelling.

The Ekman pumping velocity associated with eddy-wind interactions is another mechanism that could play a key role in dipole development and evolution. Under persistent Tehuano wind conditions, the vertical velocities inside the cyclonic vortex may reach ~ 0.4 m d^-1^ (upwelling) or a net displacement of ~ 2 m during one Tehuano wind event, while the vertical velocity of the anticyclonic eddy may reach ~ 2 m d^-1^ or a net displacement of ~ -7.5 m during one Tehuano wind event.

The typical magnitude reported for Ekman pumping that includes eddy-wind interaction is ~0.1 m d^-1^ [33], which is consistent with the magnitudes reported here (in the absence of Tehuano winds). Vertical velocities intensify under Tehuano wind conditions, where the linear component is dominant over the non-linear Ekman pumping component. Since the effect of the wind on thermocline depth can be obtained via the Ekman pumping velocity, we can deduce that under Tehuano wind conditions, the thermocline deepens (lifts) in anticyclonic (cyclonic) eddies, favoring the growth of asymmetrical vortices, which could respond to the asymmetry of the wind jet, as explained below. In the absence of these winds, the observed pattern inverts. A lifting (sinking) of the thermocline in anticyclonic (cyclonic) eddies has been reported in the literature [35–37] that tends to attenuate the eddy.

The inertial trajectory of the Tehuano winds and absolute vorticity could explain the asymmetry of Ekman pumping inside the dipole vortices. Ekman pumping is stronger inside anticyclonic eddies than cyclonic vortices.

Over the GT, the winds follow an inertial trajectory, turning westward after leaving the coast and gradually adjusting to a near geostrophic balance around 10°N and becoming east winds [6]. The wind trajectory imprints larger (negative) wind stress curl on the west side of the GT compared to the (positive) wind stress curl on the east side. Consequently, there is a preference for the production of negative relative vorticity, as has been reported for the coastal region [42]. Therefore, the strengthening of the Ekman downwelling inside anticyclonic vortices can be explained by an increase in negative wind stress curl on the vortex and by a reduction of the absolute vorticity (f + ζ), strengthening downwelling. For the cyclonic vortex, Ekman upwelling was inhibited by increased absolute vorticity. These factors contributed to an understanding of the asymmetry in Ekman pumping inside the vortices of the dipole.

During anticyclonic vortex propagation, other Tehuano wind events may take place that re-intensify Ekman downwelling by the mechanism described above. This effect could extend to the vortices up to ~200 km offshore (.i.e., during two or three weeks), as has been reported by Chang et al. [30], who observed the re-intensification of the vortex at the sub-seasonal timescale of the sea surface height. During the wind weakening stage, the wind stress curl decreases and consequently so does Ekman pumping.

The role of the linear and non-linear components with regard to the Ekman pumping velocity was assessed. Our results indicated that the linear component was the main component in net Ekman pumping (Figs 8 and 9). Far from the direct influence of Tehuano winds, a feature of Ekman pumping is the intensification of Ekman upwelling (downwelling) inside anticyclonic (cyclonic) eddies, which may be associated with the influence of eddy shape or wind direction. Based on satellite observations and numerical studies, Li et al. [39] found that upwelling pumping was enhanced (reduced) when the wind was parallel (perpendicular) to the extension of the anticyclonic vortex. If we assume a vortex takes the form of an ellipse, the major axis must thus be oriented in the same direction as the wind.

For the cyclonic vortex, the influence of other Tehuano winds could weaken it. Once the Tehuano wind ceases, the vortex propagates westward. In general, the intermittency between Tehuano winds lasts between 3 and 10 days [5,8,34]. Therefore, the cyclonic vortex should propagate westward to the central region of the GT, as was observed with vortex C1 (Fig 3B) and vortex C3 (Fig 5D and 5E). The effect of successive Tehuano wind events on the vortex could induce Ekman downwelling along the west flank of the cyclonic vortex, which implies a weakening of the vortex, in addition to the exposure of the vortex to the jet stream generated under the wind. Recently, Hong et al. [44] evaluated the impact of the surface heat flux and wind stress on surface cooling in the GT. They found that the surface wind stress induces more cooling in the mixed layer under the Tehuano wind via upwelling associated with Ekman divergence at the surface. This effect produces more upwelling (downwelling) to the east (west) side of the jet stream region, while the impact of the surface heat flux on the ocean is limited within the mixed layer (~30 m). These results support our assumption that the cyclonic vortices can be eroded by their exposure to the effects of Tehuano winds.

Recently, Amedo-Repollo et al. [45] documented the generation and evolution of a seasonal cyclonic vortex generated by gap wind jets, where the estimated vertical velocities were Ο [15] m d^-1^, based on direct measurements and wind fields that were derived from satellite products. Such thermocline displacements were consistent with the hydrographic structure of the vortex. Amedo-Repollo et al. [45] point out that the time-integrated cumulative effect of wind stress curl played a key role in the generation of cyclonic vortex and a similar mechanism could be present on the eastern coast of the GT. Under Tehuano wind conditions, the cyclonic eddy is structured and maintained on the eastern side of the gulf. With the relaxation of Tehuano winds, the vortex moves westward exposing itself to the influence of other Tehuano winds that generate strong jet currents (~1 m s^-1^) that can erode the vortex, a process that has not yet been analyzed and that is relevant to oceanographic dynamics and biological processes. For example, the transport of physical properties from the cyclonic eddy to the anticyclonic vortex can impact its thermohaline structure and as a consequence the dynamics of the vortex. This structure can effectively advect the properties of the GT toward the interior of the ocean, as suggested by dispersion pattern of drifters.

Under certain conditions, the cyclonic eddy develops and propagates. Based on satellite images, Müller-Karger and Fuentes-Yaco [15] suggested that cyclonic vortex formation was most likely to occur when a period of low-intensity winds followed a shorter, strong wind event. In this study, we have documented the generation and propagation of a dipole in the absence of Tehuano winds. The anticyclonic eddy propagated southwestward and the cyclonic eddy westward due to the beta effect and self-advection [38,41]. However, the cyclonic eddy may be attenuated by coastline-eddy interactions. These interactions could be another factor that inhibits the production of the cyclonic eddy in the GT.

The triggers and evolution of dipoles in the GT were documented from June to October in 2000. Although the analysis covers a relatively short period, our research strongly suggests that Tehuano wind events may have pronounced effects on dipoles in the GT by promoting the surface attenuation of the cyclonic eddy and the strengthening of the anticyclonic eddy. While the eddy-wind interactions intensify the upwelling (downwelling) inside the cyclonic (anticyclonic) vortex, the downwelling inside the anticyclonic vortex is stronger than the upwelling inside the cyclonic vortex, which could reflect asymmetrical wind forcing. Since the Tehuano winds over the gulf play a crucial role in the dynamics [9–12,14], biological productivity [16,21–23], and the ocean-atmosphere flux of the CO_2_ [24] of the GT, more detailed and extensive analysis on the influence of the Tehuano winds in the GT should be directed toward advancing our understanding of the complex dynamics of the gulf and the effects of these winds on marine ecosystems. Studies have already been proposed to analyze ocean-atmosphere interactions coupled with numerical modeling [46,47].

## Conclusions

This study provides information on the generation and evolution of dipoles generated and subsequently modified by Tehuano winds. The wind effect inside vortices was evaluated via Ekman pumping. Tehuano winds can generate a dipole from pre-existing cyclonic or anticyclonic circulation. For pre-existing cyclonic circulation, the Tehuano wind event confines and intensifies the cyclonic circulation on the east side of the gulf, while on the west side, an anticyclonic vortex develops. In the wind relaxation stage, dipole vortices propagate. The cyclonic vortex, in its journey to the west, may enter the central region of the Gulf of Tehuantepec. At this point, another Tehuano event may occur and erode the vortex by a jet current induced by the wind, while the anticyclonic vortex propagates freely and intensifies with Tehuano wind events.

Under Tehuano wind conditions, Ekman pumping velocities showed a dipolar structure in the Gulf of Tehuantepec. A downwelling area was present on the west coast and an upwelling area was present on the east coast, which is a pattern that promotes the development of dipoles. Tehuano winds produced downwelling (upwelling) inside anticyclonic (cyclonic) eddies. For anticyclonic (cyclonic) eddies, net vertical velocities could reach ~ -2.0 (0.5) m d^-1^ during the most intense period of a Tehuano wind event and decrease during the wind relaxation stage. Once the Tehuano winds ceased, upwelling (downwelling) predominated inside anticyclonic (cyclonic) eddies, with vertical velocities of ~ 0.1 (~ -0.1) m d^-1^.

The vertical transport of nutrients from the subsurface to the surface can take place within the cyclonic eddies associated with Ekman suction. In the Gulf of Tehuantepec, this vertical pumping to the surface may be intensified by eddy-Ekman pumping. The subsequent rupture of the cyclonic vortex by Tehuano wind events could disperse nutrients through the jet stream generated by Tehuano winds and could also transfer nutrients (tracers) into the anticyclonic vortex as suggested by the dispersion pattern of the drifters.

## Supporting information

S1 Fig The circulation and wind fields before the triggering of the first dipole. (TIF)

S2 Fig The circulation and wind fields before the triggering of the second dipole. (TIF)

S3 Fig The circulation and wind fields before the triggering of the third dipole. (TIF)
