# Supplementary figures and images for: Dipole-wind interactions under gap wind jet conditions in the Gulf of Tehuantepec, Mexico: A surface drifter and satellite database analysis
Source: PLoS One. 2019 Dec 23;14(12):e0226366. doi: 10.1371/journal.pone.0226366 (PMC6927792; doi:10.1371/journal.pone.0226366)

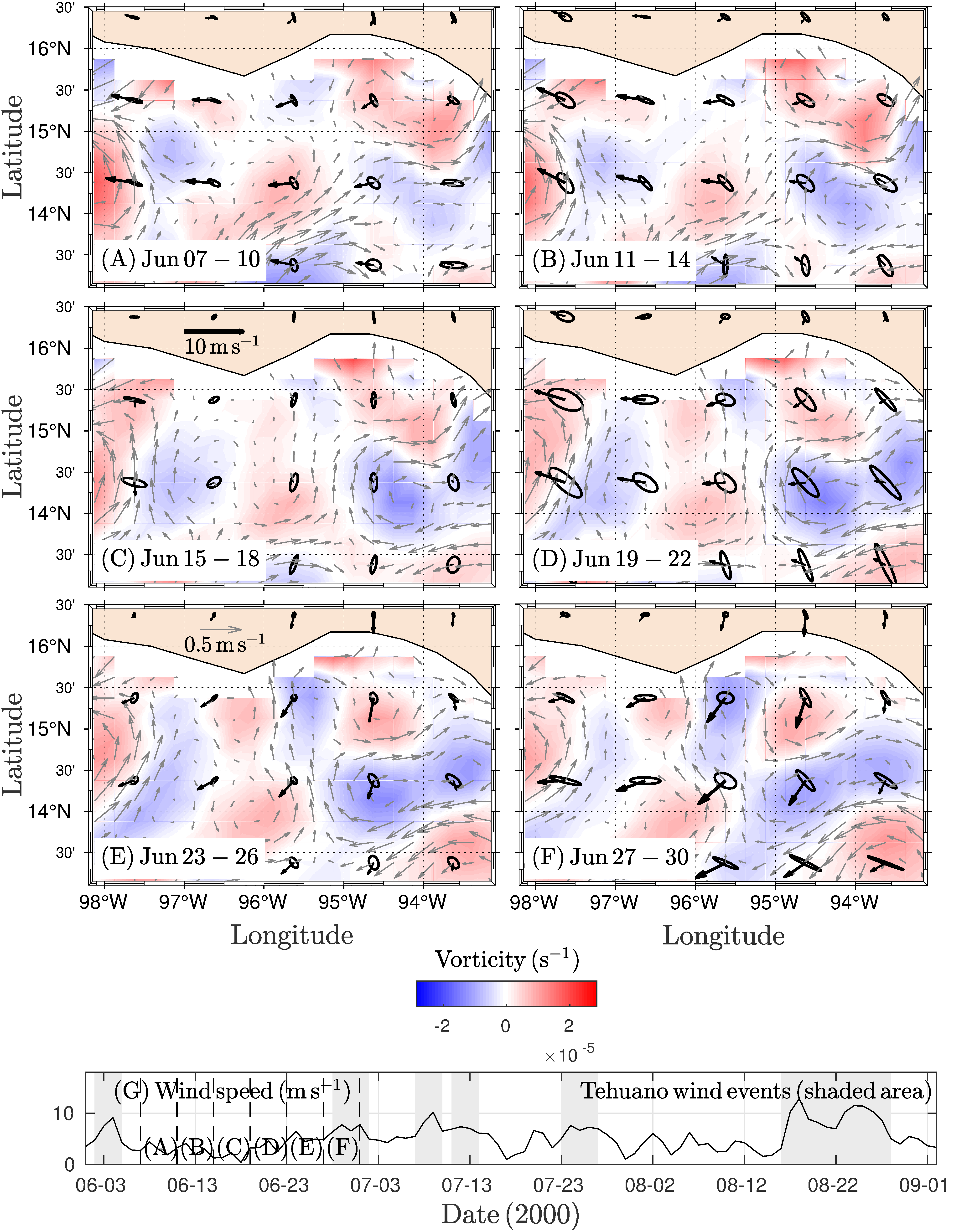

Supplement: S1 Fig — (TIF) [file pone.0226366.s001.tif]

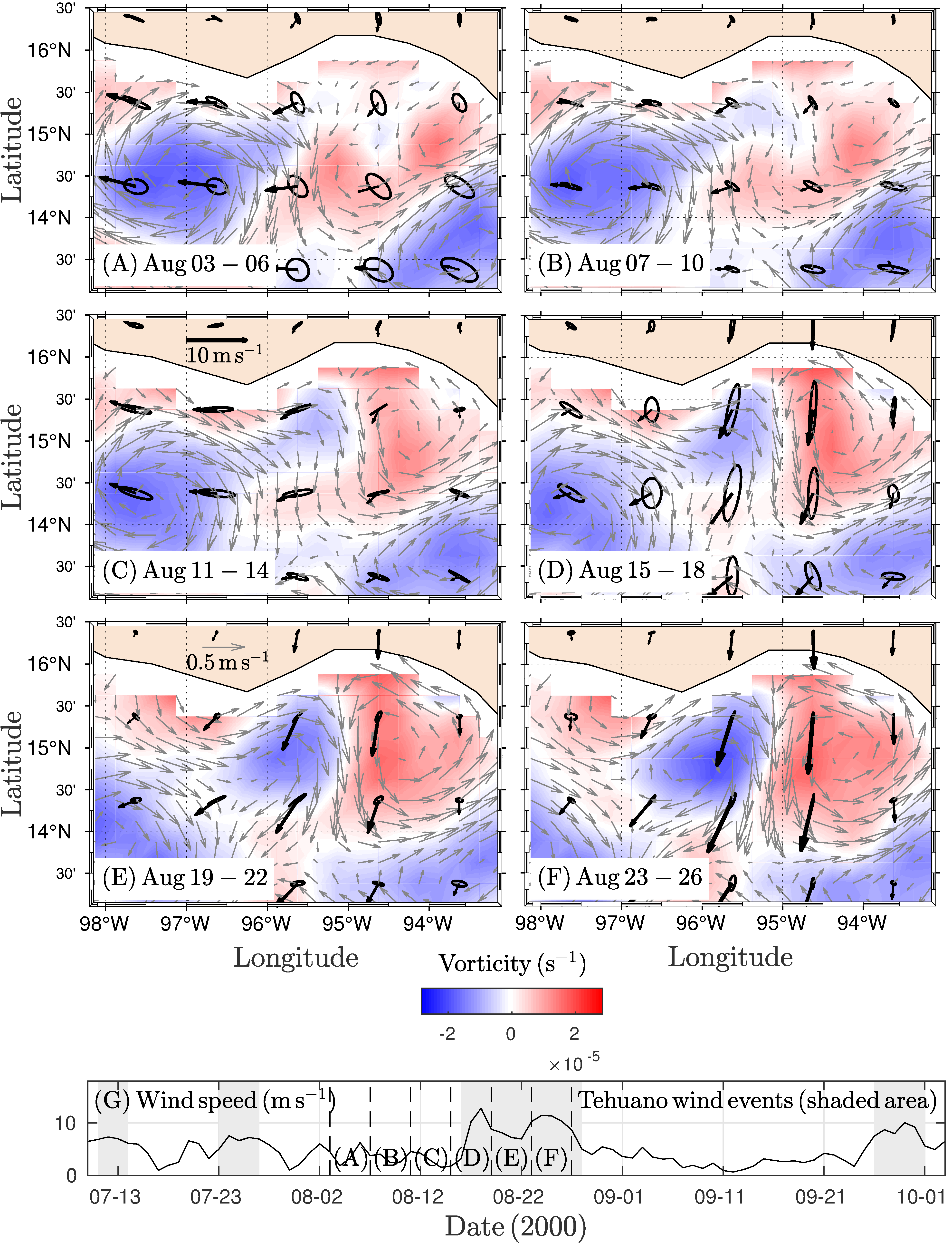

Supplement: S2 Fig — (TIF) [file pone.0226366.s002.tif]

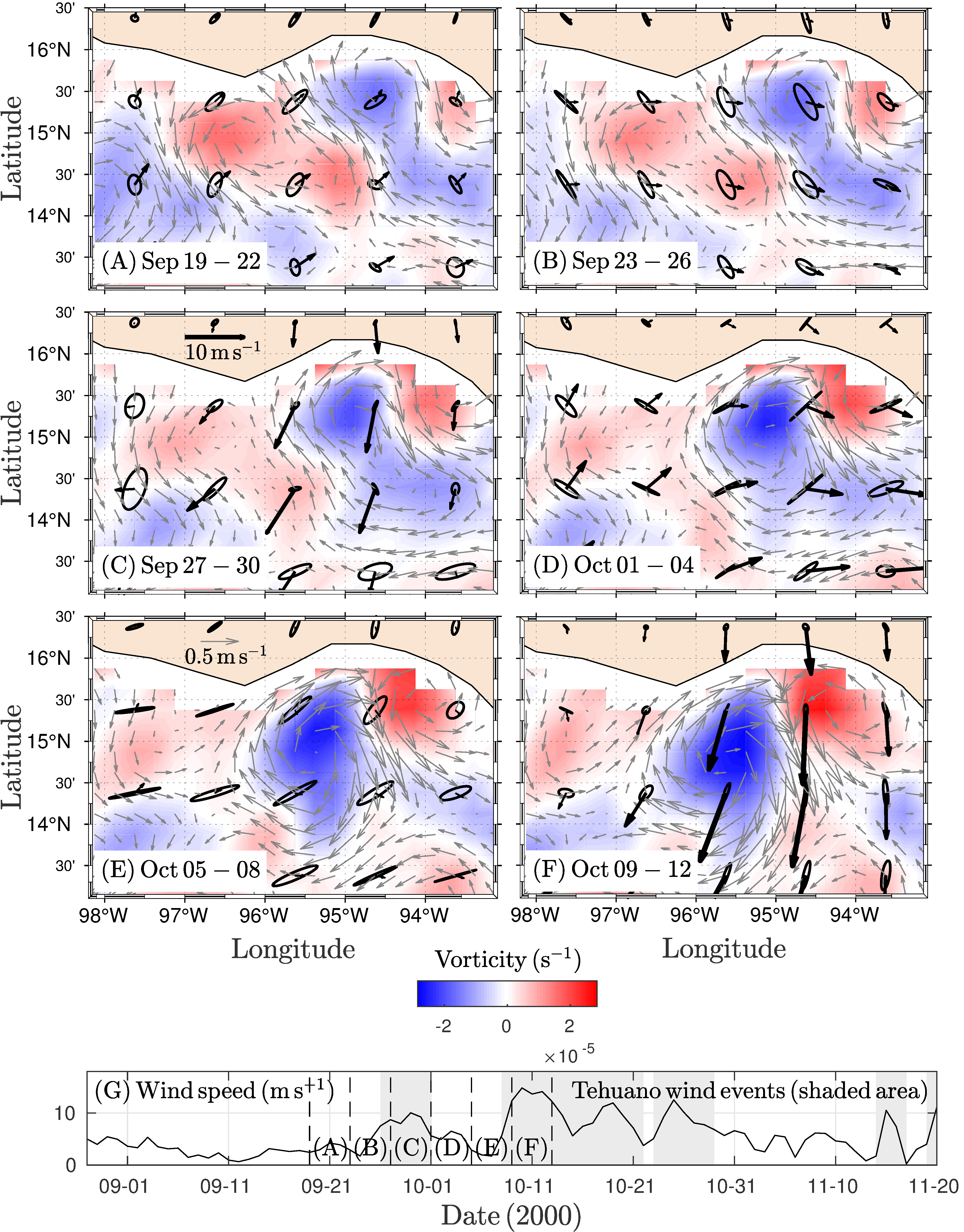

Supplement: S3 Fig — (TIF) [file pone.0226366.s003.tif]
